# The Absence of the AtSYT1 Function Elevates the Adverse Effect of Salt Stress on Photosynthesis in Arabidopsis

**DOI:** 10.3390/ijms23031751

**Published:** 2022-02-03

**Authors:** Miroslav Krausko, Zuzana Kusá, Darina Peterková, Mária Labajová, Ajay Kumar, Andrej Pavlovič, Michaela Bačovčinová, Martin Bačkor, Ján Jásik

**Affiliations:** 1 Department of Experimental Plant Biology, Institute of Botany, Plant Science and Biodiversity Center, Slovak Academy of Sciences, 845 23 Bratislava, Slovakia; miroslav.krausko@savba.sk (M.K.); kusa.zuzana@savba.sk (Z.K.); darina.peterkova@savba.sk (D.P.); maria.labajova@savba.sk (M.L.); ajay.kumar@savba.sk (A.K.); 2 Department of Biophysics, Faculty of Science, Palacký University, 783 71 Olomouc, Czech Republic; andrej.pavlovic@upol.cz; 3 Department of Botany, Institute of Biology and Ecology, Faculty of Science, Pavol Jozef Šafárik University, 041 54 Kosice, Slovakia; michaela.bacovcinova@upjs.sk (M.B.); martin.backor@upjs.sk (M.B.); 4 Department of Biochemistry and Biotechnology, Institute of Biotechnology Faculty of Biotechnology and Food Sciences, Slovak University of Agriculture, 949 76 Nitra, Slovakia

**Keywords:** *Arabidopsis thaliana*, SYNAPTOTAGMIN 1, salt stress, photosynthesis, stomata

## Abstract

*Arabidopsis thaliana* SYNAPTOTAGMIN 1 (AtSYT1) was shown to be involved in responses to different environmental and biotic stresses. We investigated gas exchange and chlorophyll *a* fluorescence in Arabidopsis wild-type (WT, ecotype Col-0) and *atsyt1* mutant plants irrigated for 48 h with 150 mM NaCl. We found that salt stress significantly decreases net photosynthetic assimilation, effective photochemical quantum yield of photosystem II (Φ_PSII_), stomatal conductance and transpiration rate in both genotypes. Salt stress has a more severe impact on *atsyt1* plants with increasing effect at higher illumination. Dark respiration, photochemical quenching (qP), non-photochemical quenching and Φ_PSII_ measured at 750 µmol m^−2^ s^−1^ photosynthetic photon flux density were significantly affected by salt in both genotypes. However, differences between mutant and WT plants were recorded only for qP and Φ_PSII_. Decreased photosynthetic efficiency in *atsyt1* under salt stress was accompanied by reduced chlorophyll and carotenoid and increased flavonol content in *atsyt1* leaves. No differences in the abundance of key proteins participating in photosynthesis (except PsaC and PsbQ) and chlorophyll biosynthesis were found regardless of genotype or salt treatment. Microscopic analysis showed that irrigating plants with salt caused a partial closure of the stomata, and this effect was more pronounced in the mutant than in WT plants. The localization pattern of AtSYT1 was also altered by salt stress.

## 1. Introduction

Unlike animals, plants are sessile organisms that must deal with an ever-changing environment from which they cannot escape. They have developed diverse survival mechanisms, and plenty of genes, including plant synaptotagmins (SYTs) that help them to last in adverse conditions have been identified [1]. Plant SYTs are orthologues of animal SYTs, well-known calcium sensors that participate in the release of neurotransmitters at the synapses of their neuronal systems [2], and recently discovered extended SYTs (E-SYTs) [3]. PSYTs contain, similarly to classical SYTs, an N-terminal transmembrane sequence and two closely spaced C2 domains and an additional synaptotagmin-like mitochondrial-lipid binding protein (SMP) domain, which is characteristic of E-SYTs. E-SYTs contain three or five tandem C2 domains [3].

Arabidopsis has a small synaptotagmin family that is comprised of six members [4,5], and among them, *AtSYT1* is ubiquitously, and the most abundantly expressed [6]. Protein was initially found to be enriched in the insolubilized fraction during cold acclimation [7]. Later, mutant and knockdown *atsyt1* seedlings were demonstrated to be hypersensitive to damage by salt [6] and cold [8]. It was also proposed that AtSYT1 facilitates adaptive responses to mechanic [9] and ionic stress [10]. Further study revealed that AtSYT1-overexpressed plants exhibit reduced membrane lipid peroxidation, upregulated heat shock transcription factors, and heat shock proteins [11]. AtSYT1 is also involved in the response to pathogen attacks. The participation of AtSYT1 in the movement of different viruses between cells through plasmodesmata is well documented [12,13,14,15,16]. Finally, AtSYT1 negatively controls immune secretory pathways to powdery mildew fungi infection by regulating the steady-state levels of the plasma membrane (PM) syntaxin PEN1 [17]. AtSYT1 protein is localized in the PM and the endomembrane system [6,8,12], and in particular, it is enriched at endoplasmic reticulum (ER)–PM contact sites (ER–PM CS) [9,10,13,18,19].

The exact mechanisms by which AtSYT1 is involved in the various processes are not established in detail. Pioneering studies disclosed the involvement of the protein in maintaining the PMintegrity by resealing it after damage by salt and cold [6,8]. Recent studies revealed the crucial function of AtSYT1 in tethering cortical ER and PM. It should stabilize the ER network at ER–PM CS [9,10,18,19]. Salt stress causes an increase in AtSYT1 abundance at ER–PM CS and their expansion [10]. AtSYT5 [20] and AtSYT3 [21] homologs are also present at ER–PM CS and these three proteins are likely functionally redundant there. The current view is that the N-terminal transmembrane domain of AtSYT1 and other AtSYTs anchor protein to the ER, C2 domains ensure Ca^2+^ dependent ER–PM tethering and an SMP domain transfers lipids between the PM and the ER. Abiotic stress such as salinity causes PM instability by accumulating diacylglycerol (DAG) and SYTs as tethers between the ER, and the PM could help maintain PM homeostasis by transporting DAG to the ER [10,21].

Soil salinization is a problem in many countries, and crop loss due to salinity is an increasing threat to agriculture worldwide [22]. All critical physiological processes in plants are directly or indirectly affected by salt stress, and among them, photosynthesis is generally significantly impaired by this stressor [23]. Here we present data demonstrating that the absence of the *ATSYT1* gene function increases the adverse effect of salt treatment on this crucial process in the model plant organism, *Arabidopsis thaliana*.

## 2. Results

### 2.1. Gas Exchange and Chlorophyll a Fluorescence Measurements

**Figure 1:**
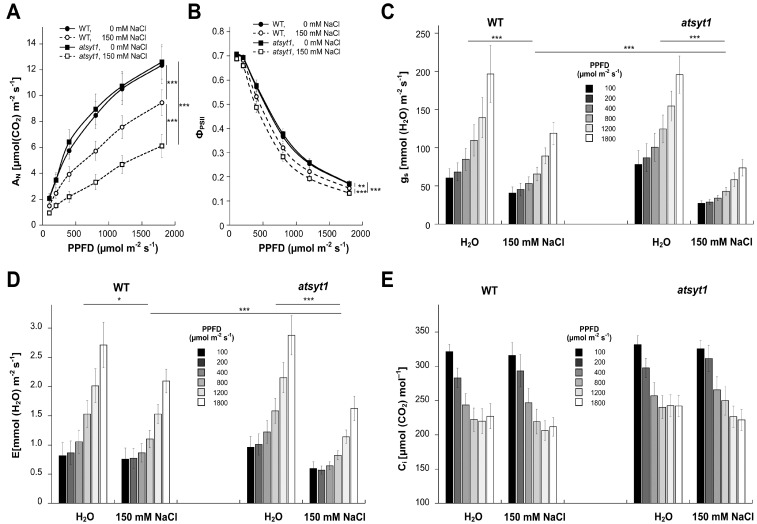
Light response curves for gas exchange and chlorophyll *a* fluorescence parameters in leaves of wild-type (WT) and *atsyt1* plants irrigated with water and 150 mM NaCl. (**A**) Net photosynthetic rate (A_N_). (**B**) The effective photochemical quantum yield of photosystem II (Φ_PSII_). (**C**) Leaf stomatal conductance (g_s_). (**D**) Transpiration rate. (**E**) Internal CO_2_ concentrations (c_i_). Data are shown as means ± SE (*n* = 8 for plants irrigated with water and *n* = 12 for plants treated with 150 mM salt). Effects of photosynthetic photon flux density (PPFD) in combination with effects of genotype or salt treatment were estimated by a two-way ANOVA test. PPFD showed a statistically highly significant effect (*p* ≤ 0.001) in all cases. Asterisks denote significant effects of genotype or salt treatment (* *p* < 0.05, ** *p* < 0.01, *** *p* < 0.001).

**Figure 2:**
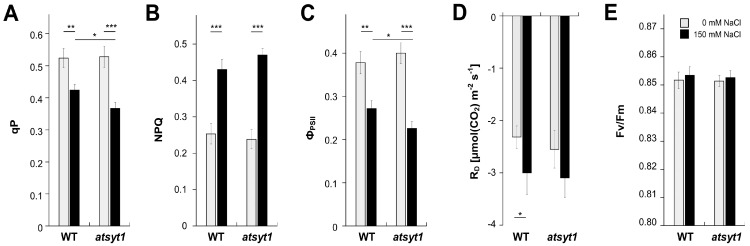
Dark respiration (R_D_) and chlorophyll *a* fluorescence parameters in leaves of wild-type (WT) and *atsyt1* plants irrigated with tap water and 150 mM NaCl under constant 750 µmol m^−2^ s^−1^ photosynthetic photon flux density (PPFD). (**A**) Coefficients for photochemical quenching (qP). (**B**) Coefficients for non-photochemical quenching (NPQ). (**C**) Effective photochemical quantum yields of photosystem II (Φ_PSII_). (**D**) Dark mitochondrial respiration (R_D_). (**E**) Maximal quantum yields of photosystem II (F_v_/F_m_). Asterisks denote significant differences between groups assessed by *t*-test (* *p* < 0.05, ** *p* < 0.01, *** *p* < 0.001). Data are shown as means ± SE (*n* = 8 for plants irrigated with water and *n* = 12 for plants irrigated with 150 mM salt).

In previous studies on AtSYT1, salt stress was estimated exclusively on seedlings growing under in vitro conditions, but experiments on advanced plants are lacking. Salt stress profoundly reduces the efficiency of photosynthesis; therefore, we wished to verify if AtSYT1 has a role in the tolerance to salt stress in regard to this essential physiological process in green plants. We investigated gas exchange and chlorophyll *a* fluorescence in wild-type (WT) and *atsyt1* plants watered for 48 h with 150 mM NaCl and compared them with plants irrigated with tap water. The measurement of the gas exchange in WT and mutant plants revealed no significant differences in the light response curves of the net photosynthesis (A_N_) to increasing photosynthetic photon flux density (PPFD) when the soil was soaked with tap water (Figure 1A). Salt treatment significantly decreased A_N_ in both genotypes compared to untreated plants with a more substantial impact on *atsyt1* than WT plants, mainly at higher illumination (Figure 1A). The light response curve of effective photochemical quantum yield of photosystem II (Φ_PSII_) (Figure 1B), which was measured simultaneously with gas exchange, matched well with decreased A_N_ in response to salt stress. Lower Φ_PSII_ was also significantly connected with *atsyt1* mutation. Again, no differences were observed between WT and *atsyt1* plants when irrigated with tap water. Stomatal conductance (g_s_, Figure 1C) and rate of transpiration (E, Figure 1D) increased with increasing PPFD, and for both parameters, no differences were revealed between WT and *atsyt1* plants when the soil was moistened with tap water. However, salt stress reduced g_s_ and E at each PPFD value, and a significant difference occurred between WT and mutant plants. The increases in the g_s_ and E when the light intensity increased were more inhibited in *AtSYT1* gene-defective plants than in WT (Figure 1C,D). The intercellular CO_2_ concentration (c_i_) decreased with increasing illumination in all cases, and no significant differences were recorded regardless of salt treatment or genotype (Figure 1E). Other chlorophyll *a* fluorescence parameters were measured at 750 µmol m^−2^ s^−1^ PPFD. The values for photochemical quenching (qP, Figure 2A), non-photochemical quenching (NPQ, Figure 2B), Φ_PSII_ (Figure 2C) and rate of respiration (R_D_, Figure 2D) were affected by salt treatment in both WT and *atsyt1* plants. Significant differences between mutant and WT plants under salt stress were only recorded for qP and Φ_PSII_. The maximum photochemical quantum yields of photosystem II (F_v_/F_m_) remained stable regardless of genotype or salt treatment (Figure 2E).

### 2.2. Photosynthetic Protein Abundance

**Figure 3:**
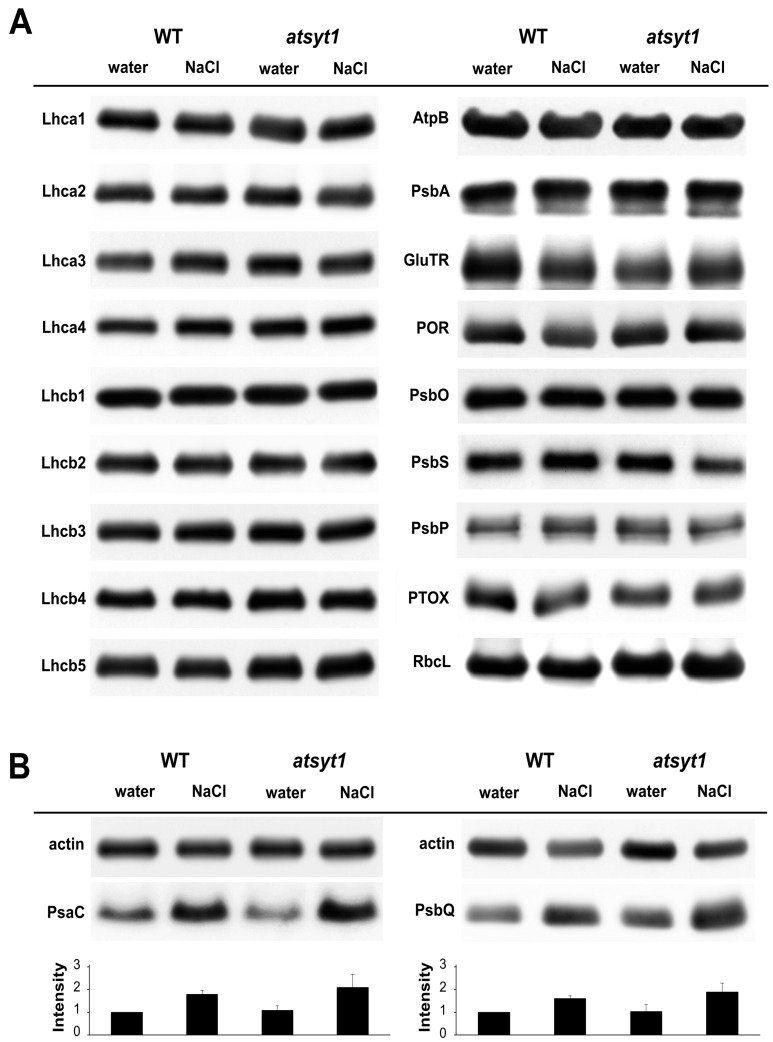
The abundance of photosynthetic proteins in leaves of wild-type (WT) and *atsyt1* plants irrigated with tap water or NaCl solution at 150 mM. (**A**) Proteins of which abundances were not affected by the absence of AtSYT1 and NaCl treatment. (**B**) Proteins with increased abundances after irrigation with the salt solution. All experiments were repeated two to four times with similar results. Actin was used as an internal loading control. Intensities of bands were quantified using ImageJ. The values obtained for target proteins were divided by the values for actin for that sample and normalized to the WT irrigated with water. Columns in the graph represent the means of four repetitions of the blots, and bars represent SE.

Since atsyt1 plants were more sensitive to the toxic effect of salinity in regard to photosynthesis, we tried to find the reason of the lower tolerance of the *atsyt1* mutant. Photosynthesis is a complex physiological process with many proteins directly involved or participating in the assemblage of photosynthetic protein complexes. In a comprehensive Western blot analysis, we studied the abundance of 20 representative proteins with divergent functions in photosynthesis. We could not find any differences in most estimated proteins (Figure 3A). Only PsaC, a peripheral subunit of the photosystem I complex, and PsbQ, a component of the oxygen-evolving complex of photosystem II, were enriched in extracts derived from salt-stressed plants. Nevertheless, only PsbQ was slightly more abundant in mutant than WT plants (Figure 3B) T when irrigated with the salt solution for 48 h.

### 2.3. Stomata Behavior

**Figure 4:**
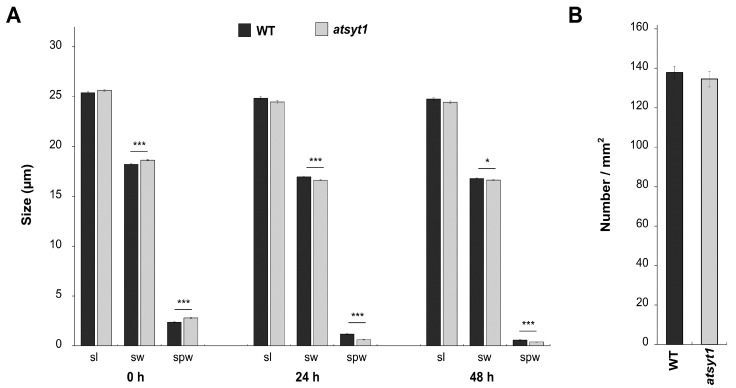
Effect of salt on stomata parameters in WT and *atsyt1* plants. (**A**) Stomata length (sl), width (sw), and pore width (spw). (**B**) Densities of stomata. Asterisks denote significant differences between groups assessed by *t*-test (* *p* < 0.05, *** *p* < 0.001). Data are shown as means ± SE (*n* ≥ 500 in (**A**), *n* = 100 in (**B**)).

Carbon dioxide is an essential substrate for photosynthesis, and the stomata control its uptake. A_N_ correlates with g_s_, and as we have found both to be more affected in *atsyt1* than in WT plants under salt stress (Figure 1C), we analyzed stomata behavior after salt treatment. At first, we studied stomata density, and we did not find a statistically significant difference between mutant and WT plants (Figure 4B). When we compared the stomata lengths, these were similar in both genotypes when plants were irrigated with tap water (Figure 4A). We also did not notice differences between genotypes in the stomata distribution pattern in the leaf epidermis. The stomata become shorter in plants treated with salt, but there was also no difference between mutant and WT plants. The stomata and stomata pore widths were somewhat larger in *atsyt1* than WT plants when the soil was soaked with tap water (Figure 4A). Moistening the plants with the salt caused a partial closure of the stomata, and this effect was more pronounced in the mutant than in WT plants (Figure 4A).

### 2.4. Content of Pigments

**Figure 5:**
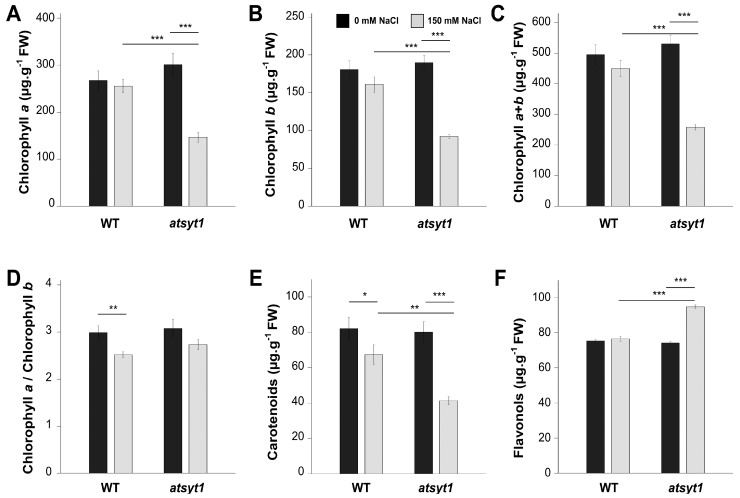
Pigments contents in leaves of salt-stressed wild-type (WT) and *atsyt1* plants. (**A**) Chlorophyll *a*. (**B**) Chlorophyll *b*. (**C**) Total amounts of chlorophylls. (**D**) Chlorophyll *a* to chlorophyll *b* ratio. (**E**) Carotenoids. (**F**) Flavonols. Asterisks denote significant differences between groups assessed by *t*-test (* *p* < 0.05, ** *p* < 0.01, *** *p* < 0.001). Data are shown as means ± SE (*n* = 45 in (**A**–**E**), *n* = 15 in (**F**)).

In the next step, we measured the pigment content, namely, chlorophylls, flavonols and carotenoids. In WT plants, we observed a slight but not significant reduction in the amount of chlorophyll *a* (Figure 5A), chlorophyll *b* (Figure 5B) and total chlorophylls (Figure 5C) after salt treatment. However, a decrease in chlorophyll contents in salt-stressed *atsyt1* plants was significant compared with salt-treated WT plants or untreated *atsyt1*. Salt stress resulted in a decreased ratio of chlorophyll *a* to chlorophyll *b*, but the difference was statistically significant only for WT plants (Figure 5D). The abundance of carotenoids was significantly reduced in salt-treated WT plants, and in *atsyt* plants, this decrease was statistically highly significant (Figure 5E). Flavonols showed the opposite trend, and their content was increased significantly in leaves of salt-stressed mutant plants (Figure 5F).

### 2.5. AtSYT1 Dynamics in Guard Cells

**Figure 6:**
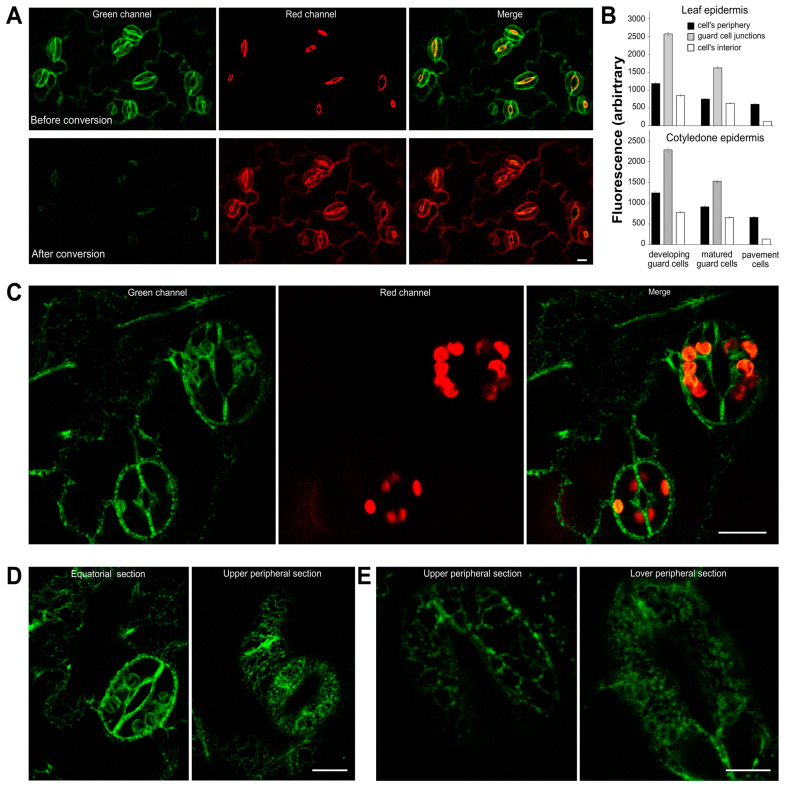
AtSYT1 in guard cells. (**A**) The red signal observed before photoconversion in guard cells of AtSYT1-Dendra2 plants and the green signal visible after photoconversion represent autofluorescence. Bar = 10 µM. (**B**) Fluorescence signal intensities in epidermal cells of seedling leaves of AtSYT1-GFP and cotyledons of AtSYT1-Dendra2. Data are shown as means ± SE (*n* ≥ 150). (**C**) AtSYT1-GFP in leaf epidermal cells. The red signal is emitted by chloroplast. Bar = 10 µM. (**D**) AtSYT1-GFP in guard cell of the seedling leaf. Bar = 10 µM. (**E**) AtSYT1-GFP at the upper and lower peripheries of the guard cells in the seedling leaf treated by 150 mM NaCl for 24 h. Bar = 5 µM.

As we found that stomata react profoundly to NaCl treatment, we wished to verify if the distribution of AtSYT is also affected by salt in stomata guard cells. We examined cotyledons of 5-day-old AtSYT1-Dendra2 seedlings and leaves of 10-day-old AtSYT1-GFP seedlings growing in vitro. Using the equatorial optical section, we found that the signal emitted by AtSYT1-Dendra (cotyledons) and AtSYT1-GFP (leaves) was prominent in guard cells at their periphery and especially enriched at the junctions between the stoma guard cells (Figure 6A,B). Pavement cells’ peripheries showed lower signal intensities than guard cells (Figure 6B). The green signal was also observable inside the cells, especially around chloroplasts (Figure 6C). In a photoconvertible experiment, we also noted that the fluorescence signal surrounding the pore was an unspecific autofluorescence (Figure 6A). When we estimated the localization pattern of AtSYT1 on optical sections through the top and bottom of guard cells, we observed a densely punctate appearance that was distinguishable from adjacent cells in untreated plants (Figure 6D). In seedlings treated with 150 mM salt for 24 h, the signal in the upper periphery of guard cells shows the reticular shape. However, in the bottom section, we observed a densely punctate arrangement (Figure 6E).

## 3. Discussion

Originally, AtSYT1 was shown to participate in membrane resealing in the roots of the model organism *Arabidopsis thaliana* under salt and cold stress [6,8]. However, later studies have documented that the role of this protein in stress responses is more complex [9,10,11,12,13,17,19]. Only seedlings that are several days old and growing in vitro have been analyzed in previous studies. In the present study, we examined more advanced plants grown in soil. It is well known that salt stress profoundly reduces the efficiency of photosynthesis [24]; therefore, we wished to verify if AtSYT1 has a role in the tolerance to salt stress with regard to this process. We note here that the absence of AtSYT1 impacts the deteriorating effect of salinity on photosynthesis, a crucial physiological process occurring in plants. We considered the results of the previous high-throughput phenotyping study by Awlia et al. [25] in the design of our experiments, and treated plants in an intensive growth period with 150 mM NaCl for two days to find differences between genotypes. We have established, as expected, that salt-stressed plants display lower photosynthesis efficiency in regard to almost all of the investigated parameters under salt stress. Importantly we also found that *atsyt1* plants are more sensitive to salt stress regarding photosynthesis than WT plants.

Salinity is well known to affect photosynthetic capability in many plant species, and potential reasons have been intensively discussed [24,26,27,28,29,30,31].

Generally, the salt stress may decrease the A_N_ due to its osmotic and toxic effects and through stomatal and non-stomatal modes. Stomatal limitation occurs shortly after the stress manifests itself in the plant’s physiological response [32]. Stomatal behavior alone could be responsible for a two-fold decrease in the photosynthesis rate in the light [33,34]. A prolonged stress period or combination of stresses usually result in non-stomatal limitation through photoinhibition of PSII or inactivation of some enzymes in the Calvin–Benson cycle [35,36,37]. We found common reactions to salt stress observed in different plant species previously, such as decreasing A_N_, g_s_, E, qP, Φ_PSII_ or increasing NPQ (Figure 1 and Figure 2) [38,39,40,41,42]. On the other side, c_i_ and F_v_/F_m_ were not affected (Figure 1E and Figure 2E). The unchanged F_v_/F_m_ indicates that the salt stress used in this study did not cause significant PSII damage in both genotypes. F_v_/F_m_ values were also stable in many different species when exposed to mild salt stress for a few days [43,44,45]. The activation of NPQ (Figure 2B) in response to salt stress probably sufficiently dissipates excess excitation energy without significant damage of PSII in both genotypes. Therefore, decreased Φ_PSII_ is likely a consequence of decreased A_N_ and g_s_, which are tightly coupled [32]. In accordance with the gas exchange data (e.g., g_s_), we observed significantly more closed stomata in salt-treated plants (Figure 4A). It is well known that stomata secure CO_2_ acquisition and prevent desiccation. Salinity causes stomata closure and mesophyll conductance suppression, and accordingly, A_N_ may decrease due to CO_2_ restraint [26]. However, increased duration of salt stress may also inactivate enzymes in the Calvin–Benson cycle [37]. Because c_i_ values were unaffected by salt stress despite lower g_s_ (Figure 1C,E), salt stress might also inhibit some enzymes in the Calvin–Benson cycle. Relative invariability in c_i_ in response to environmental stress conditions has been observed formerly (e.g., [46]) and indicates the existence of a stomata–photosynthesis coupling mechanism.

The inhibition of A_N_, g_s_, E and Φ_PSII_ by salt stress were significantly stronger in *atsyt1* plants (Figure 1). We believe that different stomatal behavior, the sensitivity of the Calvin–Benson reactions and decreased chlorophyll content may be partly behind the lower photosynthetic performance of the *atsyt1* mutant. It is well known that optimal stomatal function depends on the patterning of stomata, and the size and density of stomata determine the maximum g_s_ [33]. We estimated all these parameters, and we did not observe any differences between the mutant and WT plants. This finding suggests that both genotypes show similar g_s_ when growing under standard conditions. However, when we analyzed stoma apertures, we observed differences in the extent of the response to salt between genotypes. In fact, *atsyt1* showed a more reduced stomata aperture after 24 h salt treatment than WT plants and mutant stomata were more closed also after 48 h of treatment than WT stomata in accordance with the parameter of g_s_ (Figure 1C and Figure 4A). Because significant inhibition of g_s_ in *atsyt1* plants did not affect c_i_ values (Figure 1C,E), the increased salt-stress sensitivity of Calvin–Benson cycle enzymes may also be considered in *atsyt1*. Indeed, lower qP values (Figure 2A) in *atsyt1* salt-treated plants indicate increased excitation pressure at PSII as a result of decreased consumption of electrons by the Calvin–Benson cycle due to increased enzyme inhibition or decreased CO_2_ diffusion. However, increased excitation pressure did not cause significant PSII damage in *atsyt1* plants as F_v_/F_m_ indicates (Figure 2E).

Salt stress affects the expression of many genes. Recent proteomic studies offer plenty of examples of up- and down-regulation of proteins related to photosynthesis. There is also evidence of salts’ effect on the abundance of proteins assessed in the present study by Western blotting assay. Several reports provide information about the increasing abundance of oxygen-evolving complex subunits. Particularly, PsbP protein was enriched in extracts of different plant species subjected to salt stress [47,48,49,50,51,52,53]. Nevertheless, in oat leaves, its amount decreased in response to salinity [42]. Also, PsbO was up-and downregulated [42,49,54,55], and PsbQ was upregulated in mulberry [56]. Several proteomic studies have recognized the effect of salt on the abundance of light-harvesting complex proteins from both Lhca [47,57,58] and Lhcb [47,50,52,56] subfamilies. ATP synthase beta subunits [48,49,51,55,58] and the RuBisCO large subunit [48,52,55,58] were shown to be regulated in both directions. The inconsistency in the results may be explained by the influence of many factors such as salt concentration, duration of salt treatment, plant salt sensitivity, genotype and co-application of other protective stimuli with salt.

We evaluated the abundance of twenty essential proteins acting as subunits of various complexes participating in photosynthesis in a comprehensive Western blot assay. With two exceptions, we could not determine significant differences between the two genotypes and between salt-stressed and unstressed plants. PsaC and PsbQ were enriched in salt-stressed plants (Figure 3B). PsbP, which was frequently found to be upregulated in different plants [47,48,49,50,51,52,53], was 20% more abundant in salt-stressed WT plants than unstressed samples in our experiments, but we found no differences for *atsyt1*. Likely, in our case, photosynthesis was not reduced owing to the diminished amount of protein participating in this process. Probably, 48 h of salt treatment was too short to cause significant changes in the abundance of studied proteins.

Photosynthetic pigment analysis may also partially explain the reduced photosynthetic capacity in salt-treated *atsyt1* plants (Figure 5). During photosynthesis, chlorophyll captures the energy from light, carotenoids have photoprotective and an accessory light-harvesting function [59], and flavonols are antioxidants [60]. It is well known that photosynthetic pigments may be damaged by different stresses, which result in the reduced light-absorbing efficiency of PSI and PSII photosystems [61]. For many plant species, salt stress was shown to decrease the chlorophyll content due to increased levels of the toxic Na^+^ cation [40,50,62,63,64,65]. A decrease in carotenoids in salt-stressed plants was also reported [66,67]. It is tempting to assume that the decreased amount of chlorophyll in salt-stressed plants in our experiments was due to their breakdown rather than reduced biosynthesis. In fact, we could not find differences in the abundance of key regulatory enzymes involved in the chlorophyll biosynthesis pathway: glutamyl-tRNA reductase 1 (abbreviated as GluTR in Figure 3A) and protochlorophyllide oxidoreductase (abbreviated as POR in Figure 3A).

Finally, we estimated the abundance and localization of AtSYT1 using protein fluorescence tags, GFP and Dendra2 in guard cells of stomata. AtSYT1 has been recently intensively studied in epidermal cells of cotyledons, and its essential function has been proposed at ER–PM CS [9,10,18,19]. We found AtSYT1-GFP and AtSYT1-Dendra2 to be enriched at the periphery of guard cells compared to the surrounding pavement cells, and in particular, fusion proteins accumulated at the junctions between the two guard cells (Figure 6A,B). AtSYT1 was also apparent in the endomembrane system of guard cells (Figure 6C,D). The abundance of AtSYT1 in guard cells indicates that AtSYT1 plays an important role in guard cell function.

Interestingly, the stomata in *atsyt1* were more closed under salt stress than in WT plants, and as a consequence, g_s_ was also significantly reduced in the mutant. Different stimuli regulate stomatal closure and opening, and vesicular trafficking in guard cells plays an essential role in these processes [68]. The participation of animal SYTs in vesicular trafficking is well known [69], and a similar role was suggested for AtSYT1 in Arabidopsis roots under salt stress [6]. The guard cells control stomata closing and reopening, and many plasma membrane proteins or proteins associated with the plasma membrane are involved in this process [70]. Logically, plasma membrane proteins need to be speedily recycled through membrane trafficking as stomata respond rapidly to changing environments. There is good evidence for slowing stomatal reopening due to reduced recycling of the KAT1 K^+^ channel from endosomal membranes to the plasma membrane in *syp121* mutant [71]. The KAT1 K^+^ channel is responsible for K^+^ uptake by the guard cells, and SYP121, a SNARE member, is an essential player in vesicular trafficking. It was suggested that water flux and the transport of other solutes in guard cells might be directly coordinated with membrane trafficking [70]. AtSYT1 may be directly involved in recycling essential plasma membrane proteins that regulate guard cell contraction and expansion. An indirect mode of AtSYT1 action is also possible as AtSYT1 down-regulates the abundance of SYP121, which is also known as PEN1, possibly through endocytosis (17). Additionally, the role of AtSYT1 in delivering the membranes through exocytosis to the plasma membrane in speedily expanded guard cells or membrane removing from the plasma membrane through endocytosis during guard cells shrinkage should be considered. Finally, the participation of AtSYT1 and AtSYT3 in removing toxic DAG from the plasma membrane under salt stress, as suggested recently by Ruiz-Lopez et al. [21], could play an important role in very active guard cells.

In conclusion, AtSYT1 does not seem to be essential for plants surviving in favorable growing conditions, although it is a more abundantly and ubiquitously expressed member of the SYT family in Arabidopsis [6]. For example, *atsyt1* seedlings show unaffected phenotype in vitro when grown on a standard medium, and reduced root growth compared to control seedlings was observed only under salt stress [6]. As demonstrated in the present study, *atsyt1* and wild plants show almost identical photosynthesis parameters when growing in the soil. However, in the mutant plants, responses to sudden salt stress were much stronger in almost all parameters than in WT plants. Recent studies suggest that some AtSYTs may have a redundant function [20,21]. Perhaps less abundantly expressed AtSYT3 or AtSYT5 may provide a substitute AtSYT1 function when plants grow in a suitable environment. However, the AtSYT1 function seems to be essential for plants to respond efficiently to different stresses.

## 4. Material and Methods

### 4.1. Plant Material, Cultivation Conditions and Salt Treatment

Seeds of *Arabidopsis thaliana*, ecotype Col-0 (wild-type, WT) and *atsyt1-2* mutant (SAIL_775_A08, [6], referred to here as *atsyt1*) were sown in pots (4.5 cm deep with a radius of 6 cm) in a soil substrate (50% peat moss, 30% perlite, 20% sand). Pots with WT and *atsyt1* plants were kept in the cultivation room and randomly distributed on shelves under standard conditions: a 16/8-h light/dark cycle, with a light intensity of 150 µmol m^−2^ s^−1^, photosynthetic photon flux density (PPFD), a temperature of 22 (±0.5) °C and air humidity of 41–55%. Plants were regularly irrigated with tap water. After ten days, surplus plants were removed, and one plant per pot was kept for experiments. Plants were grown in standard conditions for another three weeks. For all experiments, we used healthy plants, approximately at the stage of inflorescence emergence as characterized by Boyes et al. [72]. Two days before measurements and sample collection, pots were immersed in 150 mM NaCl dissolved in tap water for 30 min to saturate the soil and left to drain for 10 min before being returned to the cultivation shelf. The same procedure was repeated 24 h later. The control plants were treated similarly with tap water. Plants stressed with 150 mM NaCl for 48 h were used for gas exchange, chlorophyll *a* fluorescence analysis and microscopic stomata parameters assessment. The stomata were also assessed after 24 h of treatment. For microscopy analysis, we used AtSYT1-Dendra2 and AtSYT1-GFP lines as described previously [6,73,74]. Seeds were surface sterilized with 70% ethanol and 1.5% (*v*/*v*) sodium hypochlorite and germinated on the ½MSMO medium (#M6899, Merck KGaA, Darmstadt, Germany) supplemented with 1% (*w*/*v*) sucrose. The medium was solidified with seven g/L agar-agar (#05039, Merck KGaA, Darmstadt, Germany), and media pH was adjusted to 5.7. For seedling cultivation, we used Petri dishes with a diameter of 10 cm. Petri dishes were kept in a growth chamber at 21 °C and 100 μmol m^−2^ s^−1^ PPFD continuous light. After five and ten days, seedlings were moved onto the new medium supplemented with 150 mM NaCl for 24 h.

### 4.2. Simultaneous Measurement of Gas Exchange and Chlorophyll a Fluorescence

To determine the photosynthetic gas exchange and chlorophyll *a* fluorescence, we used an infrared gas exchange analyzer Ciras-2 (PP Systems, Hitchin, UK) connected to PLC6 leaf cuvette with attached Fluorcam FC1000LC camera (Photon Systems Instruments, Brno, Czech Republic). WT and *atsyt1* plants irrigated with NaCl or tap water for 48 h before measurement were initially adapted to dark for 30 min, and then a single leaf of plant was enclosed in the PLC6 leaf cuvette. Minimal fluorescence (F_0_) was quantified using weak light of 0.1 µmol m^−2^ s^−1^ PPFD, λ = 455 nm and then maximum fluorescence (F_m_) was measured with a saturation pulse of 3000 µmol m^−2^ s^−1^ PPFD, 800 ms duration, λ = 620 nm. The ratio variable/maximum fluorescence (F_v_/F_m_) was calculated as (F_m_ − F_0_)/F_m_. The induction curve of photosynthesis was observed for 15 min after turning on an actinic light of intensity of 750 µmol m^−2^ s^−1^ PPFD. Ten regularly spaced saturation pulses (3000 µmol m^−2^ s^−1^ PPFD, 800 ms duration, λ = 620 nm) were triggered during induction measurement to calculate the quenching coefficient. The actinic light was then turned off, and F_0_′ was measured. The parameters Φ_PSII_, qP and NPQ were calculated according to Maxwell and Johnson [75]. The same enclosed leaf was used to measure the light response curves. After a short relaxation period, until R_D_ was measurable, the irradiance of 70 µmol m^−2^ s^−1^ PPFD was turned on. After stabilization, the light intensity was increased stepwise (100, 200, 400, 800, 1200 and 1800 µmol m^−2^ s^−1^ PPFD), and the measurement took 4 min in each step. At the end of each intensity period, the saturation pulse (3000 µmol m^−2^ s^−1^ PPFD, 800 ms duration, λ = 620 nm) was applied to determine Φ_PSII_. Thus, the light response curves of A_N_, E, g_s_ and Φ_PSII_ were recorded simultaneously. The concentration of CO_2_ during the whole procedure was set to 400 ppm, leaf temperature was kept at 23 ± 1 °C, and relative air humidity was between 50–55%. After the measurements were taken, the actinic light was turned off, and after 10 min, R_D_ was measured for 5 min. The leaf area enclosed in the cuvette was determined using calibrated Fluorcam software. The obtained A_N_, R_D_, E and g_s_ values were recalculated to account for area differences. Together, eight plants irrigated with tap water and twelve plants watered with a saline solution were used for each genotype.

### 4.3. Stomata Density and Size Analysis

For stomata analysis, fully expanded rosette leaves were collected from plants watered with tap water or 150 mM salt solution for 24 and 48 h. Abaxial sites were immediately coated with a thin layer of clear nail polish. After drying, the polish film was peeled from leaves, moved onto a slide and covered by a coverslip. Five images were captured from each polish replica using an Olympus FV1000 confocal laser scanning microscope (Olympus, Tokyo, Japan) equipped with Levenhuk M1400 PLUS Microscope Digital Camera (Levenhuk, Inc., Tampa, FL, USA) and associated LevenhukLite software. Stomata density was counted over the entire area of the image captured by UPlanFLN 10×/0.30 objective, which was 0.2622 mm^2^. Four leaves from each of five plants were collected, so a hundred images were analyzed per WT and *atsyt1* seedlings. For the analysis of stomatal dimensions, images were captured using UPlanSApo 60×/1.35 Oil objective, and the stomata length and width, and pore width of at least 500 stomata were measured using the ImageJ program (NIH, Bethesda, MD, USA).

### 4.4. Western Blot Assay

Total protein extraction was done using a modified EZ procedure [76]. Briefly, 300 mg leaf samples snap-frozen in liquid nitrogen were homogenized in 1.5 Eppendorf microtubes with 1 mL E-buffer (200 mM Tris-HCl pH 8.8, 10% glycerol, 1% SDS, Sigma-Aldrich Protease Inhibitor Cocktail, P9599, Merck KGaA, Darmstadt, Germany) for plant cell and tissue extracts) by a metal micro pestle using a hand drill. The extract was centrifuged for 20 min at 16,000× *g* and 4 °C, and then 800 µL of supernatant was mixed with 200 µL Z-buffer (12% SDS, 10% glycerol, 22% β- mercaptoethanol, 0.001% bromophenol blue). All chemicals were obtained from Merck KGaA (Darmstadt, Germany), unless otherwise stated. Protein concentration was determined by the DC Protein assay kit (Bio-Rad Laboratories, Hercules, CA, USA) according to the manufacturer’s instructions. All samples were adjusted to the same protein concentration with EZ buffer (E buffer to Z buffer ratio 8:2). After denaturation at 95 °C for five min, proteins were separated with SDS-PAGE using the Mini-PROTEAN^®^ Tetra electrophoresis system (Bio-Rad) and Tris-Gly buffer (25 mM Tris, 192 mM Gly, and 0.1% SDS, pH 8.3). Ten micrograms of protein was loaded per lane. 4% polyacrylamide (Acrylamide/Bis Solution, 29:1, Bio-Rad) in buffer containing 125 mM Tris, 0.1% SDS, 0.1% APS and 0.01% TEMED, pH 6.8 was used in stacking and 8 to 15% polyacrylamide in buffer consisting of 375 mM Tris, 0.1% SDS, 0.1 APS% and 0.01% TEMED, pH 8.8 in the resolving layer. Separated proteins were transferred onto an Immun-Blot^®^ PVDF Membrane (Bio-Rad) employing a Mini Trans-Blot^®^ Cell system (Bio-Rad) and transfer buffer (25 mM Tris-base, 192 mM glycine, 0.1% (*w*/*v*) SDS, 20% methanol, pH 8.3). The membrane was blocked in TBST buffer (20 mM Tris-HCl, pH 7.4, 180 mM NaCl, and 0.1% Tween 20) with 5% skim milk ((#42590, SERVA Electrophoresis GmbH, Heidelberg, Germany) for one h and then probed for 1 h with primary antibodies diluted in TBS supplemented with 5% skimmed milk. After that membranes were washed three times with TBST for 10 min and incubated with appropriate secondary antibody in TBST buffer with 5% skim milk for 1 h. After washing with TBST (three times for 10 min) and TBS (two times 5 min), the Clarity Western ECL Substrate (#1705061, Bio-Rad) was applied onto a membrane, and the light signal from the membrane was captured on photographic paper, which was developed and fixed in the normal way. Membranes were probed with antibody in dilution as follows: Anti-PsbA (D1) (Agrisera, Vännäs, Sweden, AS05 084, 1:20,000), Anti-RbcL (Agrisera, AS03 037, 1:10,000), anti-Lhca1 (Agrisera, AS01 005, 1:15,000), Anti-Lhca2 (Agrisera, AS01 006, 1:25,000), Anti-Lhca3 (Agrisera, AS01 007, 1:7500), Anti-Lhca4 (Agrisera, AS01 008, 1:5000), Anti-Lhcb1 (Agrisera, AS01 004, 1:2000), Anti-Lhcb2 (Agrisera, AS01 003, 1:20,000), Anti-Lhcb3 (Agrisera, AS01 002, 1:8000), Anti-Lhcb4 (Agrisera, AS04 045, 1:7000), Anti-Lhcb5 (Agrisera, AS01 009, 1:6000), Anti-PsbO (Agrisera, AS06 142-33, 1:10,000), Anti-PsbP (Agrisera, AS06 167, 1:6000), Anti-PsbQ (Agrisera, AS06 142-16, 1:7500), Anti-PsbS (Agrisera, AS09 533, 1:12,000), Anti-GluTR (Agrisera, AS10 689, 1:5000), Anti-POR (Agrisera, AS05 067, 1:2000), Anti-PTOX (Uniplastomic, Biviers, France, AB012, 1:2000), Anti-PsaC (Agrisera, AS10 939, 1:1000), Anti-AtpB (Agrisera, AS05 085, 1:5000). The protein loading control was performed with a monoclonal anti-actin antibody (Merck KGaA, Darmstadt, Germany, A0480, 1:10,000). Goat anti-rabbit IgG:HRP (Bio-Rad, 403005, 1:10,000) and goat anti-mouse IgG:HRP (Bio-Rad, 103005, 1:10,000) were used as secondary antibodies.

### 4.5. Quantification of Chlorophylls, Carotenoids and Flavonoids

Twenty-five milligrams of fresh leaf samples were ground with 20 mg of sand and MgCO_3_ mixture (ratio 3:1) and extracted with 1 mL of 80% (*v*/*v*) chilled acetone. Then the samples were centrifuged at 8000× *g* for 5 min at a temperature of 4 °C. Chlorophylls and total carotenoids in the supernatant were determined spectrophotometrically using a Synergy HT Microplate Reader (BioTek Instruments, Inc., Winooski, VT, USA). Absorbance was measured for chlorophyll *a* at 663 nm, chlorophyll *b* at 647 nm, and carotenoids at 470 nm. A background measurement at 710 nm was subtracted from the pigment absorbance, and the data were evaluated using Gen5 software. Concentrations of assimilation pigments were calculated according to Lichtenthaler [77]. For chlorophyll and carotenoid estimations, five plants per genotype and treatment were used for the experiment, and three samples of leaves were taken for analysis from each plant. The experiment was repeated three times, so the data represented the averages of 45 samples. Total flavonoid contents were determined with the aluminum chloride colorimetric method [78]. One hundred milligrams of snap-frozen leaf material was ground, mixed with 50% methanol, and after vortexing, flavonols were extracted for two h at room temperature. Samples were then centrifuged at 10,000× *g* for 5 min. Supernatant or quercetin standards were mixed with an equal volume of 2% aluminum chloride and incubated for 60 min at room temperature. The absorbance of the reaction mixtures was measured against blank at 415 nm. The results were derived from the quercetin calibration curve, and the flavonoid content was expressed as µg quercetin equivalents (QE)/g fresh mass. Samples collected from individual plants were pooled. Five plans were used for the experiment, and the experiment was repeated three times so that the data represented the averages of 15 samples.

### 4.6. Confocal Microscopy

Five or ten-day-old seedlings were placed on a microscopic slide covered with 1.5 solidified ½MSMO medium and covered by a coverslip. Photoconversion of Dendra2 was achieved using an Olympus FV1000 confocal laser scanning microscope as described previously [73,74]. Two-channel images were acquired sequentially in the multi-track mode under the same microscope using 20X Uplan FI (0.50 NA) or 40X UPLSAPO Super Apochromat (0.90) objectives. For Dendra2 detection, the green signal was excited with a 488 nm line from an argon laser, and the signal was collected with a 505–525 nm band-pass filter. The red signal was excited with a 543 nm line from a HeNe laser, and fluorescence was collected with a 560–620 nm band-pass filter. For GFP and chlorophyll detection, excitation was done with a 488 nm line of an argon laser, and signals were collected with 505–525 nm and 610–710 band-pass filters.

### 4.7. Data Evaluation, Processing, and Presentation

Results are expressed as means ± SEM. Comparisons between salt-treated and untreated samples and *atsyt1* and WT plants were made with a two-way analysis of variance (ANOVA). Differences between WT and mutant plants in stomata parameters, chlorophyll and carotenoid contents were valued using an unpaired Student *t*-test. Graphs were prepared using the Excel program; figures were processed with the Publisher program and Photoshop software. The fluorescence signal intensities and the intensities of bands in Western blot assay were evaluated by the ImageJ package (National Institutes of Health, Bethesda, MD, USA).
